# Perioperative Outcomes Associated with Intraoperative Hypothermia in Pediatric Patients with Preserved Functional Capacity Undergoing Anesthesia: A Multivariate Analysis

**DOI:** 10.3390/jcm14207320

**Published:** 2025-10-16

**Authors:** Worachet Saezhang, Maliwan Oofuvong, Nalinee Kovitwanawong, Kanlayanee Yongyukantorn

**Affiliations:** Department of Anesthesiology, Faculty of Medicine, Prince of Songkla University, Hat Yai, Songkhla 90110, Thailand; winworachet.w@gmail.com (W.S.); nalinee.k@psu.ac.th (N.K.); springmoonlight35@gmail.com (K.Y.)

**Keywords:** hypothermia, pediatrics, temperature, perioperative, outcomes

## Abstract

**Background/Objectives**: Few studies have investigated the perioperative adverse events following intraoperative hypothermia in pediatric patients with preserved functional capacity. We aimed to assess associations between intraoperative hypothermia and adverse outcomes in pediatric patients undergoing anesthesia. **Methods**: This retrospective cohort study included children under 12 years of age who underwent anesthesia in 2020 at Songklanagarind Hospital, Thailand. Intraoperative hypothermia was defined as the occurrence of one or more episodes of a core temperature drop to <36 °C during anesthesia. Perioperative data were extracted from the hospital information system and analyzed to identify adverse outcomes. Children with an American Society of Anesthesiologists (ASA) physical status of 4–5 were excluded to ensure that only those with preserved functional capacity before surgery were included. Multivariate regression modeling was used to evaluate associations between hypothermia and adverse outcomes after adjusting for potential confounders. Odds ratios, count ratios or beta coefficients with 95% confidence intervals (CIs) were determined. **Results**: Among the 892 patients included, 169 (18.9%) experienced intraoperative hypothermia. Intraoperative hypothermia was significantly associated with postoperative ventilator requirements (*p* < 0.001), postoperative intensive care unit (ICU) admission (*p* < 0.001), longer ventilator requirements (*p* < 0.001), and prolonged ICU stays (*p* < 0.001) and hospitalization periods (*p* < 0.001). Multivariate analysis demonstrated that intraoperative hypothermia was associated with a 1.0-day longer ICU stay and a 20% higher risk of hospitalization. **Conclusions**: Intraoperative hypothermia was associated with adverse outcomes in children with preserved functional capacity undergoing anesthesia, suggesting hospital policies should be modified to ensure vigorous perioperative temperature management to mitigate these outcomes.

## 1. Introduction

The incidence of intraoperative hypothermia, defined as a drop in core temperature <36 °C, varies widely from 4% to 80% in both adults and children [1,2,3]. Most anesthetic agents induce vasodilation, leading to a decrease in patients’ core body temperatures, along with a subsequent redistribution of temperature and a decrease in the shivering threshold [4]. Pediatric patients are particularly susceptible to developing hypothermia intraoperatively owing to the immaturity of their thermoregulatory capacity and higher body surface area (BSA) relative to body weight compared to adults [5]. Boniol et al. [6] suggested that the proportion of the skin surface area in children is only similar to that of adults after reaching 12 years of age. Furthermore, the operating room environment, including extreme ambient temperatures, can influence body temperature and may lead to perioperative hypothermia from evaporation, convection, conduction, and radiation, especially in children [4]. Activation of sympathetic nervous system activity in response to hypothermia-induced increases in norepinephrine levels can also lead to increased oxygen consumption by up to 400%, especially in newborns [7]. Pearce et al. [3] also reported greater blood loss and blood transfusion requirements related to intraoperative hypothermia in infants and young adolescents up to 18 years of age. However, studies investigating the effects of perioperative hypothermia in pediatric patients are scarce [8]. Other intraoperative hypothermia-related outcomes, such as intensive care unit (ICU) admissions, prolonged hospitalization periods [9], and surgical wound dehiscence [10,11] have only been reported in adults. Therefore, we aimed to determine the incidence of intraoperative hypothermia and associated outcomes in pediatric patients aged <12 years with preserved functional capacity who underwent anesthesia.

## 2. Materials and Methods

### 2.1. Study Design and Ethical Approval

This single-center, retrospective cohort study was conducted at a tertiary care hospital located in southern Thailand. Patient demographics, clinical information, and perioperative data, as well as anesthetic records, were extracted from the hospital information system.

This study was approved by the Institutional Ethics Committee of the Faculty of Medicine, Prince of Songkla University, Thailand (approval number: REC 65-003-8-4; date of approval: 11 February 2022). The requirement for informed consent was waived owing to the retrospective nature of the study. All processes were performed in accordance with the tenets of the Declaration of Helsinki. Children younger than 12 years of age who underwent general anesthesia from 1 January 2020 to 31 December 2020 and had an American Society of Anesthesiologists (ASA) physical status of 1–3 were eligible for inclusion. Patients were excluded if they experienced at least one episode of intraoperative hyperthermia (defined as a rise in core temperature above 37.8 °C), if they underwent cardiopulmonary bypass in which intraoperative hypothermia was deliberately induced, or if no temperature monitoring was conducted intraoperatively.

### 2.2. Standard Operating Procedures for Pediatric Anesthesia

In our institution, the decision of whether to perform general anesthesia with endotracheal tube intubation or laryngeal mask airway is left to the discretion of the anesthesiology staff, as is the decision to combine epidural/caudal anesthesia with a peripheral nerve block. The selection of intravenous anesthetics or volatile agents with sevoflurane for induction can be made by either the patient or the anesthesia team. Neuromuscular blocking agents, such as rocuronium or cisatracurium, are commonly administered to facilitate intubation. During the maintenance phase, sevoflurane rather than desflurane is used as a volatile anesthetic agent to minimize the likelihood of airway complications. The core temperature is typically monitored after general anesthesia using a nasopharyngeal, esophageal, or rectal probe in cases in which the expected operation time exceeds 30 min. In the present study, temperature measurements were collected by nurse anesthetists via various routes and recorded in each patient’s anesthetic chart every 30 min (see Supplementary Figure S1 for an example). In our routine practice, core temperature is measured first unless there are specific contraindications, in which case skin, axillary, or tympanic membrane (ear probe) measurements are used. To improve accuracy, we adjusted skin/axillary temperature readings by adding 0.55 °C [12] and tympanic membrane readings by adding 0.42 °C [13] to correct the measurement closest to the core temperature. The standard operating room temperature ranges between 22 and 24 °C. A forced-air warming system was routinely used in most cases if the expected operation time exceeded 30 min.

### 2.3. Main Exposure

An intraoperative temperature of at least 36 °C was considered to be normothermia, whereas at least one episode of a core temperature falling below 36 °C indicated the occurrence of hypothermia, the duration of which was defined as the period during which the core body temperature remained below 36 °C, measured in 30 min intervals. For example, if the body temperature fluctuated from normal to below 36 °C within any 30-minute interval, 15 min was added to the total duration of hypothermia. The severity of intraoperative hypothermia in this setting was classified as mild hypothermia (34 °C to <36 °C), moderate hypothermia (32 °C to <34 °C), and severe hypothermia (<32 °C).

### 2.4. Potential Confounding Variables

The preoperative variables assessed included patient characteristics, such as body weight, height, and BSA, underlying respiratory diseases (upper and lower respiratory tract infections, allergic rhinitis, and asthma), cardiovascular disease (congenital heart disease and arrhythmia), neurological disease (epilepsy and meningitis), hematologic disease (anemia, thrombocytopenia, and coagulopathy), and endocrine disease (diabetes mellitus and hypo/hyperthyroidism), the ASA physical status, and baseline temperature. The intraoperative variables assessed included the site of surgery (superficial, eye, ear/nose/throat, abdomen, extremities, intracranial, and intrathoracic), magnitude of surgery (minor, intermediate, and major), anesthetic technique used (general anesthesia, epidural/caudal anesthesia, and peripheral nerve block), airway management approach (endotracheal tube and laryngeal mask airway), type of anesthesia administered (total intravenous anesthesia and volatile anesthesia), neuromuscular blocking agents administered (rocuronium and cisatracurium), duration of surgery, duration of anesthesia, estimated blood loss, blood transfusion requirements, and route of temperature monitoring (nasopharyngeal, esophageal, rectal, or skin sensors).

### 2.5. Definition of Variables

The BSA of each patient was calculated using the Mosteller equation [14]. The magnitude of each surgery was classified as major (surgery in which body cavities or major vessels were exposed to an ambient temperature, such as major abdominal, thoracic, major vascular, and thoracic spinal surgery with instrumentation), intermediate (surgery in which body cavities were exposed to a lesser degree, such as laparoscopic surgery, transurethral resection of the prostate, percutaneous nephrolithotomy, appendectomy, and herniotomy), and minor (superficial surgery) [15]. Active warming was defined as warming using forced air applied through partial or complete covering above or draping below the patient’s body. The duration of the operation was defined as the time from the initial incision to closure of the surgical wound. The duration of anesthesia was defined as the time from the first anesthetic drug administration to the time of extubation.

### 2.6. Outcomes

To ensure that events or adverse outcomes occurring after hypothermia were captured, the primary outcomes focused on postoperative events, including postoperative ICU admission, postoperative ventilator requirement, and reintubation. The secondary outcomes were length of hospitalization and duration of ventilator support. The other outcomes were duration of post-anesthetic care unit (PACU) stay, occurrence of nausea, vomiting, and shivering, as well as oxygen requirement.

### 2.7. Sample Size Determination

An earlier pilot study revealed an incidence of hypothermia of 18%. The required sample size was calculated using postoperative ICU admissions as the primary outcome and oxygen requirements at PACU as the secondary outcome. The percentage of patients who experienced intraoperative hypothermia and required ICU admission was 20%, whereas the percentage of patients who did not experience intraoperative hypothermia but still required postoperative ICU admission was 10%. Thus, assuming a ratio of 1:4, an alpha level of 0.05, and a power of 0.8, the required sample size was 640 patients, based on the formula for comparing proportions in a cohort study [16]. For the secondary outcome, the means (standard deviation) of duration of hospitalization of patients among hypothermic and normothermic patients were 4 (7) and 2 (8) days, respectively. Using the same assumption as for postoperative ICU admissions, the required sample size was 642 patients, based on the formula for comparing means in a cohort study [16]. However, after accounting for a potential incomplete data rate of 20%, the final required sample size was set at 803 patients, based on the larger calculation. Since 900–1000 pediatric cases are treated at our institution annually, 1 year of data collection was deemed adequate.

### 2.8. Statistical Analysis

The data were double-entered and validated using Epidata (version 3.1). All categorical variables are presented as frequencies and percentages, whereas continuous variables are presented as means and standard deviations. For intergroup comparisons of categorical variables, the Chi-square or Fisher’s exact test was used, as appropriate. The unpaired Student’s *t*-test was used for intergroup comparisons of normally distributed continuous variables; for non-normally distributed data, the Wilcoxon rank sum test was used. Since the magnitude of surgery arose from the site/type of surgery, thereby leading to high collinearity, only the site, but not the magnitude, of surgery was included in the multivariate regression modeling. Multivariate logistic regression models were used to evaluate the association between dichotomous outcomes and intraoperative hypothermia after adjusting for potential confounders. Because the durations of ICU admission and ventilator use were count data containing many zeros, negative binomial regression analysis was performed to address overdispersion for these specific outcomes, as previously described [17,18]. Hospitalization duration was log-transformed to satisfy the assumption of normality, and robust standard errors were applied to account for heteroscedasticity [19]. Predictors that were marginally associated with the outcome (i.e., those with a *p*-value < 0.2) were included in the initial multivariate regression analysis. A backward stepwise selection method was then used to identify the final model with the lowest AIC value. Hospitalization duration was reported as a count ratio with 95% confidence intervals (CIs), obtained by exponentiating the coefficients from the log-transformed model. Other associations are reported as odds ratios (ORs) or beta-coefficients (β) with 95% CIs. The threshold for statistical significance was set at *p* < 0.05.

## 3. Results

**Figure 1:**
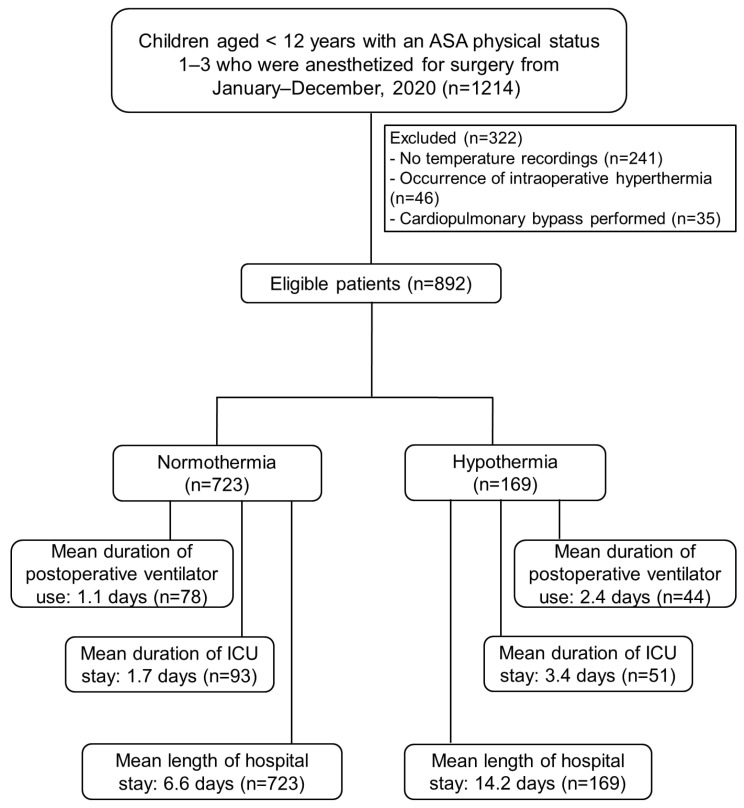
Flow diagram of participant selection. *ASA*, American Society of Anesthesiologists; *ICU*, intensive care unit.

During the period from January 2020 to December 2020, a total of 1214 patients met the inclusion criteria (Figure 1); 322 of these patients were subsequently excluded (241 did not undergo intraoperative temperature monitoring, 46 experienced intraoperative hyperthermia, and 35 underwent cardiopulmonary bypass surgery in which intraoperative hypothermia was deliberately induced). Of the 892 remaining patients, 169 (19%) experienced intraoperative hypothermia.

### 3.1. Patient Baseline Characteristics

Table 1 shows comparisons of the patients’ baseline characteristics stratified by the presence or absence of intraoperative hypothermia. Having an age < 1 year (*p* = 0.003) was the only patient-related factor significantly associated with intraoperative hypothermia. Among surgery-related factors, major surgery (*p* < 0.001) and intra-abdominal and intracranial surgery (*p* = 0.001) were significantly more common in those who experienced intraoperative hypothermia. Table 2 shows the intergroup comparisons of anesthetic techniques and intraoperative variables between those with and without intraoperative hypothermia. The intraoperative hypothermia group had a significantly higher proportion of patients who exhibited an ASA physical status of 3 (*p* = 0.005); that group also had significantly longer anesthesia times (*p* < 0.001), and there was significantly less use of neuromuscular blocking agents (*p* = 0.023) than that in the normothermia group, suggesting those factors may be associated with intraoperative hypothermia.

The intraoperative characteristics of the 169 patients with hypothermia are shown in Table 3. All patients in that group experienced mild hypothermia. The median duration of the hypothermic episodes was 60 min.

### 3.2. Hypothermia-Related Outcomes

Table 4 shows comparisons of intraoperative and postoperative variables between those who did and did not experience intraoperative hypothermia. A significantly higher percentage of patients with intraoperative hypothermia had postoperative ventilator requirements (*p* < 0.001), and that group had significantly higher postoperative ICU admission rates (*p* < 0.001) than those in the normothermia group, suggesting that those factors were significantly associated with intraoperative hypothermia. Furthermore, both the duration of the ICU stay and length of the hospitalization period were significantly longer in the hypothermia group than in the normothermia group (1.7 vs. 3.4 days and 6.6 vs. 14.2 days, respectively).

The outcomes associated with intraoperative hypothermia were further analyzed using multivariate analysis. Categorical data, such as mechanical ventilator requirements and postoperative ICU admissions, were analyzed using logistic regression. Continuous data, such as the durations of the postoperative ventilator requirement and ICU stay, were analyzed using negative binomial regression, whereas the logarithm of hospitalization duration was analyzed using linear regression.

### 3.3. Univariate and Multivariate Logistic Regression Analysis of Significant Outcomes Related to Intraoperative Hypothermia

Supplementary Table S1 shows the variables that had a *p*-value < 0.2, which were included in the initial multivariate regression analysis of significant outcomes related to intraoperative hypothermia. Table 5 demonstrates that intraoperative hypothermia was not significantly associated with either postoperative ventilator requirements or postoperative ICU admissions based on the multivariate analysis.

### 3.4. Univariate and Multivariate Negative Binomial Regression Analysis of Duration of Ventilator Requirement and ICU Stay, Related to Intraoperative Hypothermia

Supplementary Tables S2 and S3 shows the variables that had a *p*-value < 0.2, which were included in the initial multivariate regression analysis of significant outcomes related to intraoperative hypothermia. As shown in Table 6, the multivariate analysis revealed that intraoperative hypothermia significantly increased the duration of the ICU stay (1.0 days, *p* = 0.001).

### 3.5. Univariate and Multivariate Linear Regression Analysis of the Logarithm of Hospitalization Duration Related to Intraoperative Hypothermia

Supplementary Table S4 shows the variables that had a *p*-value < 0.2, which were included in the initial multivariate regression analysis of significant outcomes related to intraoperative hypothermia. As shown in Table 6, the multivariate analysis revealed that intraoperative hypothermia significantly increased the count ratio of the hospitalization period (1.2 times, *p* = 0.02).

## 4. Discussion

In the present study, the incidence of intraoperative hypothermia in pediatric patients with preserved functional capacity (ASA physical status 1–3) who underwent anesthesia was 19%, which is at the lower end of the range of values reported in other studies of 18–83% [3,20,21]. More specifically, Pearce et al. [3] reported that 52% of pediatric patients, including neonates, older children, and adolescents (aged < 18 years), experienced perioperative hypothermia, and Zhao et al. [18] reported that the rate of intraoperative hypothermia was much higher in neonates than that in infants (83% vs. 38%, respectively). Nevertheless, Hu et al. [20] demonstrated that it was possible to reduce the incidence of hypothermia in pediatric patients with burns (age < 15 years) to 18% by maintaining the temperature in the operating theater at 26–27 °C. In the setting of the present study, 98% of the children received active warming during surgery, which resulted in a lower incidence of hypothermia than that reported in other studies. In fact, all the cases of intraoperative hypothermia were classified as mild, with a drop in core temperature to 34 °C or <36 °C, compared to the 70% rate reported by Pearce et al. [3]. Previous studies have included children with a wide range of ages, from newborns to adolescents [3,22,23]. However, considering that anatomical and physiological changes in children aged > 12 years would be expected to be similar to those in adults [6,24], the present study included only children younger than 12 years of age.

Numerous adverse consequences have been reported to be associated with intraoperative hypothermia in adults in clinical settings, such as increased postoperative ICU admissions [9], prolonged postoperative hospitalization periods [8], extended PACU stays [9,25], and the occurrence of postoperative cardiac events [26,27]. Two studies have investigated intraoperative hypothermia-related outcomes in pediatric patients undergoing surgery; however, they only conducted univariate analyses [21,28]. The results of these studies showed that hypothermia was associated with postoperative ICU admissions, the length of stays in the ICU, the duration of hospitalization, the occurrence of postoperative bleeding, and transfusion requirements [21,28], as well as extended mechanical ventilation times [18]. Furthermore, Görges et al. [29] demonstrated that only wound disruption, but not surgical wound infection or blood transfusion, was a consequence of hypothermia after adjustment in a multivariate analysis in pediatric patients undergoing surgery. In the present study, a multivariate analysis was conducted in which adjustments were made for various confounders to more accurately examine the consequences of hypothermia. The findings of the multivariate analysis revealed that even mild hypothermia can lead to two important adverse outcomes, including prolonged ICU stays (1 day) and extended periods of hospitalization (1.2 times).

Hypothermia was confirmed to be an independent risk factor for specific perioperative outcomes in this study after adjusting for patient-, surgery-, and anesthesia-related factors. Since some of the patients undergoing major surgery may have had complex conditions such as pulmonary, neurologic, and congenital heart diseases that might require planned ICU admission, those underlying conditions were considered to be potential confounders and were adjusted for in the multivariate analysis; even after such adjustment, hypothermia was still significantly associated with prolonged periods of hospitalization and ICU stays (Table 6), consistent with the results reported by Oofuvong et al. [30,31]. In addition to the site of surgery (e.g., ear/nose/throat, intraabdominal, and intrathoracic)), which tended to be related to prolonged ventilator requirements, prolonged ICU stays, and hospitalization periods were also included to adjust for confounders in the multivariate analysis, and hypothermia was still significantly associated with longer ICU stays and hospitalization periods (Table 6). Adjustments were also made for patient characteristics such as age, weight, and weight-to-BSA ratio, as neonates and infants with poor nutritional status may be more susceptible to worse outcomes, including longer ventilator requirements and prolonged ICU stays and hospitalization periods, especially those who are undergoing more extensive surgery. Moreover, those patient characteristics may exacerbate hypothermia development, thereby increasing the oxygen demand, promoting arterial hypotension and pulmonary hypertension, aggravating hypoperfusion metabolic acidosis, tissue hypoxia, and ischemia, and increasing the likelihood that postoperative care will be required in the ICU [18,25].

The occurrence of postoperative cardiovascular events following the development of hypothermia has only been reported in older adults [23,24], and such events were not observed in the present study, possibly because fatal tachyarrhythmia is commonly associated with severe hypothermia (<32 °C), especially in neonates [32], and all of the patients in this study experienced only mild hypothermia. The most commonly observed arrhythmia was bradycardia, and there was no significant difference in its incidence between the hypothermic and normothermic groups. Lin et al. [33] reported that hypothermia (temperature < 35 °C) in adults being treated in a clinical setting increased the risk of reintubation, with an OR of 2.5. No studies have reported a change in reintubation rates after hypothermia in a pediatric setting, which is consistent with the present findings. In this study, reintubation was not an adverse outcome associated with hypothermia.

Two studies [3,22] showed that the amount of blood loss and blood transfusion requirements were associated with intraoperative hypothermia after undergoing extensive surgery. In this study, the proportion of patients who underwent major surgery was approximately 10%, with an average blood loss of 5 mL in both groups. Blood loss and the need for blood transfusions could be either risk factors for intraoperative hypothermia or events promoted by it, depending on the timing of the hypothermic event. In this study, univariate analyses revealed significantly greater blood loss and transfusion requirements among patients who experienced hypothermia compared with those who did not. Consequently, these variables were included in the multivariate analysis to adjust for postoperative outcomes.

The findings of this study may contribute to the limited literature regarding the consequences of intraoperative hypothermia in pediatric patients. A cost-of-illness study conducted in Australia estimated that the costs related to the treatment of inadvertent perioperative hypothermia in patients undergoing surgery exceeded one billion Australian dollars annually [34], and the adverse events reported in the present study contribute to the economic burden on individual hospitals and society as a whole. Vigorous action must be taken to lessen this burden in pediatric anesthesia settings, and the study results have several implications for achieving this goal. First, optimizing preoperative conditions by treating respiratory infections or pulmonary disease can help reduce the ASA physical status to <3 while also lessening the likelihood of poorer hypothermia-related outcomes. Second, pre-warming protocols should be implemented, and the turnover time inside operating theaters should be increased, especially for infants undergoing surgery. Third, besides active warming, an intravenous line warmer should be used, the ambient temperature should be increased, and post-procedure active warming strategies should be adopted for patients without intact skin (such as those requiring burn scrubbing). Fourth, increasing vigilance to ensure close temperature monitoring in high-risk patients can be beneficial, as can considering the optimal means of airway support for children with mild to moderate hypothermia until their core temperature has returned to normal.

One of the strengths of this study is its large sample size derived from a pool of over 1200 pediatric patients, all of whom underwent surgery within a 1-year period (2020). Furthermore, the anesthetic practices and agents administered to the patients with and without intraoperative hypothermia were similar during the study period. The amount of missing temperature monitoring data was also very low (0.45%). Finally, a multivariate analysis was conducted, with adjustment for potential confounders. Despite the positive findings, this study has some limitations. First, the retrospective nature of the study may be subject to information bias. Some data could not be collected, such as the ambient temperature of the operating room, the amount of fluid irrigation, the temperature of the fluid, and the waiting time before surgery. Core temperature was estimated using skin/axillary/tympanic membrane probes instead of being determined in the standard fashion using an esophageal/nasal/rectal probe to avoid performing invasive procedures in cases involving burns or infection. The results may also not be generalizable to other populations or settings owing to differences in routine practices across different institutions.

## 5. Conclusions

Although the rates of ventilator use and ICU admission were low among children with preserved functional capacity, mild hypothermia may still be associated with prolonged ICU stay and hospitalization, particularly among those requiring ventilator support or undergoing major surgery. Thus, maintaining normothermia during surgery should be a priority, especially in high-risk pediatric patients. Vigorous perioperative temperature management guidelines should be implemented to prevent hypothermia events and minimize the likelihood of adverse hypothermia-related outcomes.

## Figures and Tables

**Table 1:** Comparison of baseline characteristics by intraoperative hypothermia status (*n* = 892).

Variable	Intraoperative Hypothermia		*p*-Value
	No (*n* = 723)	Yes (*n* = 169)	
**Demographics**			
Male sex	468 (64.7%)	108 (63.9%)	0.8
Age (years), median (IQR)	4.6 [1.8, 7.7]	3.9 [1.2, 8.1]	0.12
Age (years) ≥1/<1	619 (85.6%)/104 (14.4%)	129 (76.3%)/40 (23.7%)	0.003 *
Body weight (kg), median (IQR)	15.6 [10.2, 22.8]	14.6 [9.0, 23.0]	0.06
Body height (cm), median (IQR)	102.0 [80.3, 122.0]	99.0 [72.0, 122.0]	0.12
Percentile weight >P3/≤P3	578 (79.9%)/145 (20.1%)	126 (74.6%)/43 (25.4%)	0.12
BSA (m^2^), median (IQR)	0.7 [0.5, 0.9]	0.6 [0.4, 0.9]	0.05
Weight-to-BSA ratio, median (IQR)	23.5 [21.1, 26.6]	23.2 [20.4, 25.6]	0.087
**Underlying disease**			
Cardiovascular disease	123 (17.0%)	26 (15.4%)	0.6
Respiratory disease	129 (17.8%)	32 (18.9%)	0.7
Neurologic disease	60 (8.3%)	30 (17.8%)	<0.001 *
Hematologic disease	220 (30.4%)	55 (32.5%)	0.6
Endocrine disease	46 (6.4%)	9 (5.3%)	0.6
**Elective/emergency surgery**	628 (86.9%)/95 (13.1%)	151 (89.3%)/18 (10.7%)	0.4
**Preoperative body** **temperature (°C), median (IQR)**	36.8 [36.6, 37.0]	36.8 [36.6, 37.0]	0.5
**Route of temperature monitoring**			0.11
Rectal	69 (9.6%)	14 (8.4%)	
Nasopharyngeal	160 (22.2%)	48 (28.7%)	
Esophageal	250 (34.7%)	54 (32.3%)	
Skin/axillary	237 (32.9%)	47 (28.1%)	
Tympanic membrane	5 (0.7%)	4 (2.4%)	
Unknown	2 (0.3%)	2 (1.2%)	
**Magnitude of surgery**			<0.001 *
Major	50 (6.9%)	30 (17.8%)	
Intermediate	510 (70.5%)	121 (71.6%)	
Minor	163 (22.5%)	18 (10.7%)	
**Site of surgery**			0.001 *
Superficial	116 (16.0%)	19 (11.2%)	
Eye, ear/nose/throat	318 (44.0%)	50 (29.6%)	
Abdomen	125 (17.3%)	38 (22.5%)	
Extremities	133 (18.4%)	45 (26.6%)	
Intracranial	17 (2.4%)	12 (7.1%)	
Intrathoracic	14 (1.9%)	5 (3.0%)	

**Note**: Data are presented as the frequency (%) or median (IQR), unless stated otherwise. * *p* < 0.05, chi-square test. *IQR*, interquartile range; *BSA*, body surface area.

**Table 2:** Comparison of anesthetic techniques and intraoperative profiles by intraoperative hypothermia status (*n* = 892).

Variable	Intraoperative Hypothermia		*p*-Value
	No (*n* = 723)	Yes (*n* = 169)	
**ASA physical status**			0.005 **
1	158 (21.9%)	39 (23.1%)	
2	377 (52.1%)	67 (39.6%)	
3	188 (26.0%)	63 (37.3%)	
**Type of GA**			
GA with caudal/epidural block	80 (11.1%)	27 (16.0%)	0.077
GA with peripheral nerve block	52 (7.2%)	18 (10.7%)	0.13
**Type of airway management**			0.2
Endotracheal tube	622 (86.0%)	139 (82.2%)	
Laryngeal mask airway	101 (14.0%)	30 (17.8%)	
**Anesthesia time (min), median (IQR)**	105.0 [80.0, 150.0]	145.0 [100.0, 190.0]	<0.001 *
**Operation time (min), median (IQR)**	70.0 [50.0, 110.0]	100.0 [60.0, 140.0]	0.001 *
**Neuromuscular blocking agent use**			0.023 ***
None	127 (17.6%)	34 (20.1%)	
Cisatracurium	595 (82.3%)	132 (78.1%)	
Rocuronium	1 (0.1%)	3 (1.8%)	
**Volatile anesthetic use**			0.019 ***
None (intravenous anesthetics only)	21 (2.9%)	1 (0.6%)	
Sevoflurane	676 (93.5%)	167 (98.8%)	
Desflurane	26 (3.6%)	1 (0.6%)	
**Active warming**	709 (98.1%)	163 (96.4%)	0.2
**Fluid use**			
Crystalloid (mL), median (IQR)	172.0 [100.0, 300.0]	163.0 [85.0, 350.0]	0.8
Colloid	7 (1.0%)	2 (1.2%)	0.7
**Blood loss (mL),** median (IQR)	5.0 [1.0, 15.0]	5.0 [2.0, 20.0]	0.011 *
**Blood transfusion**	34 (4.7%)	25 (14.8%)	<0.001 **
**Intraoperative bradycardia**	24 (3.3%)	5 (3.0%)	0.5

**Note**: Data are presented as the frequency (%) or median (IQR), unless stated otherwise. * *p* < 0.05 by Wilcoxon rank sum test; ** *p* < 0.05 by Chi-square test; and *** *p* < 0.05 by Fisher’s exact test. *ASA*, American Society of Anesthesiologists; *IQR*, interquartile range; *GA*, general anesthesia.

**Table 3:** Characteristics of the patients who experienced intraoperative hypothermia (*n* = 169).

Characteristic	Hypothermia Group
**Nadir temperature (°C)**	
Mean (SD)	35.5 (0.3)
Median (IQR)	35.6 [35.3, 35.7]
**Duration of hypothermia (min)**	
Mean (SD)	65.0 (48.6)
Median (IQR)	60.0 (30.0, 90.0)
**Severity of hypothermia**	
Mild (34 °C to <36 °C)	169 (100%)
Moderate (32 °C to <34 °C)	0

**Note**: *IQR*, interquartile range; *SD*, standard deviation.

**Table 4:** Univariate analysis of outcomes associated with intraoperative hypothermia (*n* = 892).

Variable	Intraoperative Hypothermia		*p*-Value
	No (*n* = 723)	Yes (*n* = 169)	
**Length of PACU stay (min),** median (IQR)	45.0 [35.00, 60.0]	45.0 [30.00, 60.0]	0.12
**Shivering**	6 (0.8%)	2 (1.2%)	0.7
**Nausea/vomiting**	8 (1.1%)	2 (1.2%)	0.9
**Postoperative oxygen** **requirements**	38 (5.3%)	5 (3.0%)	0.29
Oxygen cannula	7 (1.0%)	0 (0.0%)	0.4
Non-rebreather mask	2 (0.3%)	1 (0.6%)	0.2
Mask/oxygen flow	28 (3.9%)	3 (1.8%)	0.3
Oxygen box	0 (0%)	0 (0%)	
High-frequency nasal cannula	1 (0.1%)	1 (0.6%)	0.5
**Postoperative ventilator** **requirement**	78 (10.8%)	44 (26.0%)	<0.001 **
**Duration of oxygen and ventilator requirement** (days)			
Mean (SD)	1.1 (6.9)	2.4 (7.4)	<0.001 ***
Median (IQR)	0.0 [0.0, 0.0]	0.0 [0.0, 1.0]	<0.001 *
**Postoperative ICU admission**	93 (12.9%)	51 (30.2%)	<0.001 **
**Duration of ICU admission (days)**			
Mean (SD)	1.7 (8.6)	3.4 (10.1)	<0.001 ***
Median (IQR)	0.0 [0.0, 0.0]	0.0 [0.0, 1.0]	<0.001 *
**Duration of hospitalization (days)**			
Mean (SD)	6.6 (14.1)	14.2 (24.5)	<0.00 1 ***
Median (IQR)	2.0 [1.0, 5.0]	4.0 [2.0, 13.0]	<0.001 *
**Reintubation**	1 (0.1%)	2 (1.2%)	0.094

**Note**: Data are presented as the frequency (%) or median (IQR), unless stated otherwise. * *p* < 0.05 by Wilcoxon rank sum test; ** *p* < 0.05 by Chi-square test; *** *p* < 0.05 by Student’s t-test. *ICU*, intensive care unit; *IQR*, interquartile range; *PACU*, post-anesthetic care unit; *SD*, standard deviation.

**Table 5:** Multivariate logistic regression analysis of potential associations between intraoperative hypothermia and postoperative ventilator requirements or postoperative intensive care unit admissions (*n* = 892).

Variable	Postoperative Ventilator Requirement		Postoperative ICU Admission	
	Adjusted OR (95% CI)	*p*-Value	Adjusted OR (95% CI)	*p*-Value
Hypothermia	1.69 (0.72, 4.01)	0.233	1.52 (0.75, 3.05)	0.248
Age < 1 year (Ref: ≥1)	6.80 (2.07, 22.3)	0.001	6.11 (2.36, 15.8)	<0.001
Respiratory tract infection/AR/asthma	9.70 (3.55, 26.5)	<0.001	4.46 (2.19, 9.07)	<0.001
Underlying neurologic disease	6.41 (2.43, 16.9)	0.001	4.14 (1.94, 8.85)	<0.001
ASA physical status = 3 (Ref: ≤2)	26.2 (9.16, 75.0)	<0.001	9.60 (5.07, 18.2)	<0.001
Body weight (kg)	0.92 (0.86, 0.98)	0.001		
Body height (cm)			0.98 (0.97, 0.99)	<0.001
Percentile weight ≤ P3 (Ref: >P3)	0.35 (0.13, 0.98)	0.039		
Weight-to-BSA ratio < 16 (Ref: ≥16)	80.0 (15.5, 414.2)	<0.001	11.8 (3.83, 36.3)	<0.001
Major surgery	6.17 (2.14, 17.8)	<0.001	9.83 (4.35, 22.2)	<0.001
Anesthesia time (min)	1.004 (1.0003, 1.009)	0.03	1.005 (1.001, 1.009)	0.012
Emergency surgery	4.11 (1.52, 11.1)	0.005		
Intraoperative crystalloid (mL)			1.002 (1.00, 1.003)	0.051
Blood transfusion (mL)			1.004 (1.00, 1.009)	0.058

**Note:** *p*-values determined using the Wald test, *AR*, allergic rhinitis; *ASA*, American Society of Anesthesiologists; *BSA*, body surface area; *ICU*, intensive care unit; *OR*, odds ratio; *CI*, confidence interval; *Ref*, reference group.

**Table 6:** Multivariate regression analysis of associations between intraoperative hypothermia and perioperative outcomes (*n* = 892).

Variable	Duration of Ventilator Requirement (Days)		Duration of ICU Stay (Days)		Duration of Hospitalization ^¶^ (Days)	
	Adjusted β (95% CI)	*p*-Value	Adjusted β (95% CI)	*p*-Value	Adjusted Count Ratio (95% CI)	*p*-Value
Hypothermia	0.15 (−0.35, 0.66)	1	0.98 (0.47, 1.48)	0.001	1.20 (1.04, 1.36)	0.02
Age < 1 year (Ref: ≥1)	2.18 (1.39, 2.96)	0.001	2.56 (1.96, 3.16)	0.001	1.82 (1.57, 2.07)	<0.001
Respiratory tract infection/AR/asthma	1.02 (0.55, 1.50)	0.001	1.11 (0.60, 1.61)	0.001	1.16 (1.02, 1.52)	0.04
Congenital heart disease	0.36 (−0.22, 0.94)	1				
Underlying neurologic disease	2.06 (1.37, 2.75)	0.001	2.05 (1.46, 2.64)	0.001	1.27 (1.01, 1.52)	0.069
Underlying hematologic disease					1.22 (1.11, 1.34)	0.002
ASA physical status = 3 (Ref: ≤2)	2.41 (1.85, 2.97)	0.001	3.15 (2.66, 3.64)	0.001	1.51 (1.33, 1.69)	<0.001
Body height (cm)	−0.015 (−0.031, 0.00)	0.05			1.003 (1.000, 1.006)	0.048
Body weight (kg)	0.03 (−0.002, 0.058)	0.1				
Weight-to-BSA ratio < 16 (Ref: ≥16)	1.35 (0.59, 2.10)	0.01	0.73 (−0.027, 1.50)	0.1	1.30 (0.95, 1.65)	0.146
Emergency surgery	0.63 (0.073, 1.18)	0.05	0.58 (−0.003, 1.16)	0.1	1.34 (1.14, 1.54)	0.005
Major surgery			1.31 (0.74, 1.89)	0.001		
Anesthesia time (min)					1.001 (1.0006, 1.002)	0.019
Site of surgery (Ref: superficial)		0.01 *				0.001 *
Eye, ear/nose/throat	−0.74 (−1.45, 0.04)	0.05			0.85 (0.64, 1.06)	0.123
Abdomen	−1.25 (−2.01, −0.49)	0.01			1.37 (1.23, 1.69)	0.013
Extremities	−1.03 (−1.86, −0.19)	0.05			1.46 (1.28, 1.76)	0.001
Intracranial	−1.77 (−2.87, −0.66)	0.01			1.41 (0.99, 1.83)	0.1
Intrathoracic	0.19 (−0.86, 1.24)	1			2.74 (2.29, 3.19)	<0.001
Intraoperative crystalloid use (mL)	0.002 (0.000, 0.003)	0.01			1.0003 (0.999, 1.001)	0.09
Estimate blood loss (mL)	0.002 (0.000, 0.004)	0.05				

**Note: ***p*-values determined by F test statistic, * based on the likelihood ratio test. *AR*, allergic rhinitis; *ASA*, American Society of Anesthesiologists; *BSA*, Body surface area; *β*, beta-coefficient; *CI*, confidence interval; *ICU*, intensive care unit; *Ref*, reference group. **^¶^** Log-transformed.

## Data Availability

The datasets used and/or analyzed in the current study are available from the corresponding author upon reasonable request.

## Supplementary Materials

The following supporting information can be downloaded at https://www.mdpi.com/article/10.3390/jcm14207320/s1: Figure S1: Example of an anesthetic record with temperature monitoring; Table S1: Univariate logistic regression analysis of the associations between intraoperative hypothermia and postoperative ventilator requirements, postoperative ICU admissions, and reintubation (n = 892); Table S2: Univariate negative binomial analysis of the duration of ventilator requirements (*p*-value < 0.2) (n = 892); Table S3: Univariate negative binomial analysis of the duration of ICU admissions (*p*-value < 0.2) (n = 892); Table S4: Univariate linear regression analysis of the duration of log of hospitalization (*p*-value < 0.2) (*n*= 892).

## Abbreviations

The following abbreviations are used in this manuscript:

AR	Allergic rhinitis
ASA	American Society of Anesthesiologists
BSA	Body surface area
CI	Confidence interval
GA	General anesthesia
ICU	Intensive care unit
IQR	Interquartile range
OR	Odds ratio
PACU	Post-anesthesia care unit
