# Supplementary material for: Perioperative Outcomes Associated with Intraoperative Hypothermia in Pediatric Patients with Preserved Functional Capacity Undergoing Anesthesia: A Multivariate Analysis
Source: J Clin Med. 2025 Oct 16;14(20):7320. doi: 10.3390/jcm14207320 (PMC12565136; doi:10.3390/jcm14207320)
Supplement: Supplementary file 1 [file jcm-14-07320-s001.zip › Supplementary Tables.pdf]

Supplementary Table S1. Univariate logistic regression analysis of the associations between intraoperative hypothermia and postoperative ventilator requirements, postoperative ICU admissions, and reintubation (n = 892)

Variable	Postoperative oxygen requirement		Postoperative ventilator requirement		Postoperative ICU admission	
	**Crude OR (95% CI)**	**p-value**	**Crude OR (95% CI)**	**p-value**	**Crude OR (95% CI)**	**p-value**
Hypothermia	0.55 (0.21, 1.42)	0.29	3.09 (2.03, 4.7)	< 0.001	2.93 (1.97, 4.34)	<0.001
Age < 1 year (Ref: ≥1)	12.5 (8.33, 20.0)	0.007	20.0 (12.5, 33.33)	0.004	14.3 (10.0, 25.0)	0.001
Percentile weight ≤P3 (Ref: >P3)	5.45 (3.76, 7.9)	0.001	5.69 (3.78, 8.57)	0.001	4.86 (3.32, 7.12)	0.001
ASA physical status 3 (Ref: ≤2)	29.92 (18.56, 48.22)	< 0.001	34.74 (19, 63.54)	< 0.001	22.21 (13.88, 35.56)	< 0.001
Body weight to BSA ratio <16 (Ref: ≥16)	110.45 (39.29, 310.5)	< 0.001	153.8 (59.45, 397.9)	<0.001	69.35 (30.68, 156.8)	< 0.001
Body weight (kg)	0.89 (0.87, 0.91)	< 0.001	0.81 (0.78, 0.84)	< 0.001	0.88 (0.85, 0.90)	< 0.001
Body height (cm)	0.96 (0.95, 0.97)	0.001	0.95 (0.94, 0.96)	0.001	0.96 (0.95, 0.97)	0.001
Emergency surgery	4.24 (2.77, 6.48)	0.001	5.77 (3.68, 9.03)	0.002	4.12 (2.67, 6.36)	0.001
Major surgery	12.24 (7.4, 20.22)	< 0.001	11.42 (6.93, 18.82)	0.008	15.55 (9.32, 25.96)	< 0.001
Cardiovascular disease	7.05 (4.77, 10.43)	< 0.001	4.36 (2.86, 6.66)	< 0.001	4.48 (3.01, 6.68)	< 0.001
Respiratory disease	3.94 (2.69, 5.78)	< 0.001	4.81 (3.17, 7.29)	< 0.001	4.22 (2.85, 6.25)	< 0.001
Neurologic disease	3.11 (1.94, 4.96)	0.002	3.81 (2.32, 6.24)	0.003	3.82 (2.38, 6.12)	0.003
Hematologic disease	1.15 (0.8, 1.66)	0.68	1.34 (0.89, 2.01)	0.70	1.38 (0.95, 2.0)	0.32
Endocrine disease	0.66 (0.29, 1.48)	0.91	0.64 (0.25, 1.64)	0.78	0.75 (0.33, 1.68)	0.81
Site of surgery (Ref: superficial)		0.001*		0.3*		0.01*
Eye, ear/nose/throat	0.23 (0.14, 0.38)	0.006	0.56 (0.31, 1.03)	0.24	0.51 (0.29, 0.9)	0.70
Abdomen	0.52 (0.31, 0.89)	0.004	1.38 (0.74, 2.58)	0.82	1.43 (0.8, 2.55)	0.73
Extremities	0.15 (0.08, 0.31)	0.07	0.25 (0.1, 0.61)	0.21	0.35 (0.17, 0.74)	0.96
Intracranial	4.75 (2.0, 11.3)	0.80	11.6 (4.69, 28.72)	0.55	12.78 (5.04, 32.39)	0.228
Intrathoracic	11.41 (3.16, 41.25)	0.72	10.47 (3.66, 29.93)	0.94	25.97 (6.99, 96.47)	0.04
Anesthesia time (min)	1.006 (1.005, 1.008)	0.001	1.008 (1.006, 1.01)	0.001	1.009 (1.006, 1.011)	0.001
Neuromuscular blocking agent use	6.84 (2.97, 15.75)	< 0.001	5.7 (2.29, 14.21)	< 0.001	7.33 (2.95, 18.19)	< 0.001
Volatile anesthetic use	0.72 (0.21, 2.47)	0.44	1.04 (0.3, 3.56)	0.27	0.82 (0.24, 2.8)	0.38
Intraoperative crystalloid (mL)	0.997 (0.991, 1.002)	0.23	0.999 (0.996, 1.002)	0.54	1.001 (0.999, 1.004)	0.20
Estimate blood loss (mL)	1.0005 (0.999, 1.003)	0.60	0.997 (0.992, 1.003)	0.35	1.004 (1.002, 1.006)	0.51
Blood transfusion (mL)	1.009 (1.003, 1.016)	0.004	1.006 (1.004, 1.009)	0.13	1.007 (1.005, 1.010)	0.098

Note: p-values determined using the Wald test, * based on the likelihood ratio test. *ASA,* American Society of Anesthesiologists; *BSA,* body surface area; *ICU,* intensive care unit; *OR*, odds ratio; *CI*, confidence interval; *Ref*, reference group.

Supplementary Table S2. Univariate negative binomial analysis of the duration of ventilator requirements (p-value < 0.2) (n = 892)

Variable	Beta-coefficient	Standard error	p-value
Hypothermia	0.73	0.35	0.036
Age < 1 year (Ref: ≥1)	2.5690	0.3113	<0.001
Percentile weight ≤P3 (Ref: >P3)	1.23	0.33	<0.001
ASA physical status 3 (Ref: ≤2)	3.96	0.22	<0.001
Body weight to BSA ratio <16 (Ref:≥16)	2.21	0.49	<0.001
Emergency surgery	1.51	0.40	<0.001
Cardiovascular disease	1.55	0.35	<0.001
Respiratory disease	1.57	0.34	<0.001
Endocrine disease	-0.53	0.59	0.37
Site of surgery (Ref: superficial)			0.01*
Eye, ear/nose/throat	-0.39	0.39	0.32
Abdomen	0.33	0.45	0.47
Extremities	-1.12	0.46	0.01
Intracranial	0.99	0.79	0.21
Intrathoracic	2.19	0.94	0.02
Anesthesia time (min)	0.004	0.002	0.02
Intraoperative crystalloid (mL)	0.001	0.0007	0.10
Estimate blood loss (mL)	0.003	0.001	0.06
Blood transfusion (mL)	0.004	0.001	0.004

Note: p-values determined by the F test statistic, * by the likelihood ratio test. *ASA*, American Society of Anesthesiologists; *BSA*, body surface area; *Ref*, reference group.

Supplementary Table S3. Univariate negative binomial analysis of the duration of ICU admissions (p-value < 0.2) (n = 892)

Variable	Beta-coefficient	Standard error	p-value
Hypothermia	0.73	0.38	0.05
Female sex	0.17	0.31	0.58
Age < 1 year (Ref: ≥1)	2.59	0.34	<0.001
Percentile weight ≤P3 (Ref: >P3)	1.01	0.36	0.005
ASA physical status 3 (Ref: ≤2)	3.65	0.24	<0.001
Body weight to BSA ratio <16 (Ref: ≥16)	2.46	0.52	<0.001
Emergency surgery	1.36	0.43	0.002
Cardiovascular disease	1.51	0.38	<0.001
Respiratory disease	1.33	0.37	0.0004
Neurologic disease	0.56	0.49	0.25
Endocrine disease	-0.52	0.62	0.41
Site of surgery (Ref: superficial)			0.01*
Eye, ear/nose/throat	-0.32	0.36	0.45
Abdomen	1.08	0.42	0.03
Extremities	-0.50	0.48	0.30
Intracranial	0.94	0.48	0.27
Intrathoracic	2.60	0.84	0.01
Anesthesia time (min)	0.004	0.002	0.01
Intraoperative crystalloid (mL)	-0.0004	0.0007	0.62
Estimate blood loss (mL)	0.001	0.002	0.39
Blood transfusion (mL)	0.002	0.001	0.11

Note: p-values determined by F test statistic, * by the likelihood ratio test. *ICU*, intensive care unit; *ASA*, American Society of Anesthesiologists; *BSA*, body surface area; *Ref*, reference group.

Supplementary Table S4. Univariate linear regression analysis of the duration of log of hospitalization (p-value < 0.2) (n= 892)

Variable	Beta-coefficient	Standard error	p-value
Hypothermia	1.622	0.084	<0.001
Age < 1 year (Ref: ≥1)	0.80	0.09	0.005
Percentile weight ≤P3 (Ref: >P3)	1.498	0.08	<0.001
ASA physical status 3 (Ref: ≤2)	2.316	0.069	<0.001
Body weight to BSA ratio <16 (Ref: ≥16)	2.845	0.124	<0.001
Body weight (kg)	0.9902	0.002	<0.001
Body height (cm)	0.994	0.001	<0.001
Major surgery	3.665	0.108	<0.001
Emergency surgery	2.217	0.097	<0.001
Cardiovascular disease	1.475	0.088	<0.001
Respiratory disease	1.515	0.086	<0.001
Neurologic disease	1.925	0.108	<0.001
Hematologic disease	1.330	0.072	<0.001
Site of surgery (Ref: superficial)			<0.001*
Eye, ear/nose/throat	0.701	0.091	<0.001
Abdomen	1.400	0.105	0.001
Extremities	1.345	0.103	0.004
Intracranial	3.324	0.184	<0.001
Intrathoracic	4.525	0.221	<0.001
Anesthesia time (min)	1.004	0.0004	<0.001
Intraoperative crystalloid (mL)	1.0007	0.00016	<0.001
Estimate blood loss (mL)	1.003	0.00033	<0.001
Blood transfusion (mL)	1.0021	0.00031	<0.001

Note: p-values based on F test statistic, * by likelihood ratio test. *ASA*, American Society of Anesthesiologists; *BSA*, body surface area; *Ref*, reference group.
