# Supplementary figures and images for: Perioperative Outcomes Associated with Intraoperative Hypothermia in Pediatric Patients with Preserved Functional Capacity Undergoing Anesthesia: A Multivariate Analysis
Source: J Clin Med. 2025 Oct 16;14(20):7320. doi: 10.3390/jcm14207320 (PMC12565136; doi:10.3390/jcm14207320)

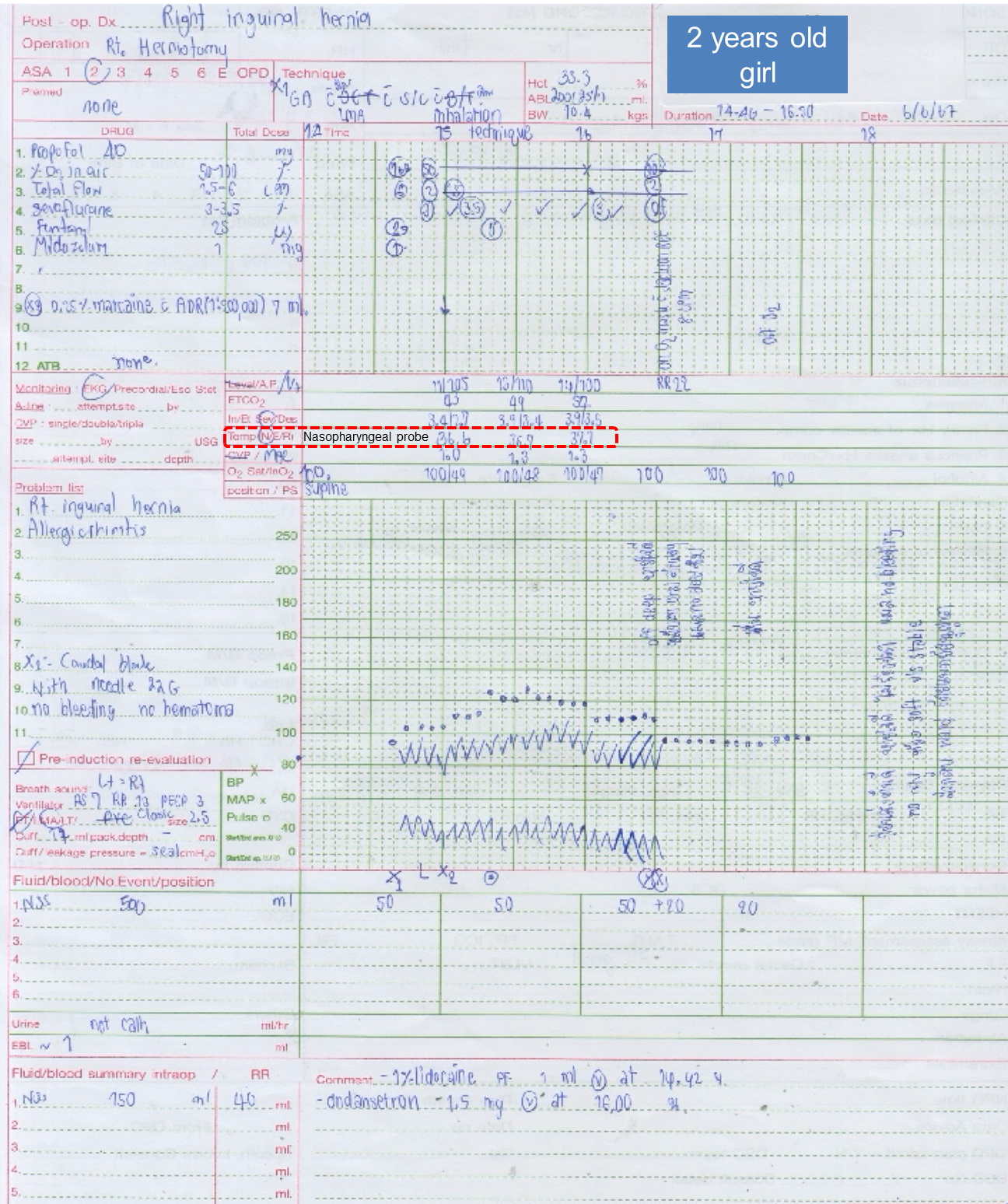

Supplement: Supplementary file 1 [file jcm-14-07320-s001.zip › Figure S1.jpg]
